# Emerging Trends in Information-Seeking Behavior for Alpha-Gal Syndrome: Infodemiology Study Using Time Series and Content Analysis

**DOI:** 10.2196/49928

**Published:** 2024-05-08

**Authors:** Jamie L Romeiser, Nicole Jusko, Augusta A Williams

**Affiliations:** 1 Department of Public Health and Preventive Medicine Upstate Medical University Syracuse, NY United States

**Keywords:** alpha-gal, alpha gal, alpha-gal syndrome, lone star tick, infodemiology, time series, content analysis, Google Trends, allergy, allergic, immune, immunology, immunological, information behavior, information behaviour, information seeking, geographic

## Abstract

**Background:**

Alpha-gal syndrome is an emerging allergy characterized by an immune reaction to the carbohydrate molecule alpha-gal found in red meat. This unique food allergy is likely triggered by a tick bite. Cases of the allergy are on the rise, but prevalence estimates do not currently exist. Furthermore, varying symptoms and limited awareness of the allergy among health care providers contribute to delayed diagnosis, leading individuals to seek out their own information and potentially self-diagnose.

**Objective:**

The study aimed to (1) describe the volume and patterns of information-seeking related to alpha-gal, (2) explore correlations between alpha-gal and lone star ticks, and (3) identify specific areas of interest that individuals are searching for in relation to alpha-gal.

**Methods:**

Google Trends Supercharged-Glimpse, a new extension of Google Trends, provides estimates of the absolute volume of searches and related search queries. This extension was used to assess trends in searches for alpha-gal and lone star ticks (*lone star tick*, *alpha gal*, and *meat allergy*, as well as *food allergy* for comparison) in the United States. Time series analyses were used to examine search volume trends over time, and Spearman correlation matrices and choropleth maps were used to explore geographic and temporal correlations between alpha-gal and lone star tick searches. Content analysis was performed on related search queries to identify themes and subcategories that are of interest to information seekers.

**Results:**

Time series analysis revealed a rapidly increasing trend in search volumes for alpha-gal beginning in 2015. After adjusting for long-term trends, seasonal trends, and media coverage, from 2015 to 2022, the predicted adjusted average annual percent change in search volume for alpha-gal was 33.78%. The estimated overall change in average search volume was 627%. In comparison, the average annual percent change was 9.23% for lone star tick, 7.34% for meat allergy, and 2.45% for food allergy during this time. Geographic analysis showed strong significant correlations between alpha-gal and lone star tick searches especially in recent years (ρ=0.80; *P*<.001), with primary overlap and highest search rates found in the southeastern region of the United States. Content analysis identified 10 themes of primary interest: diet, diagnosis or testing, treatment, medications or contraindications of medications, symptoms, tick related, specific sources of information and locations, general education information, alternative words for alpha-gal, and unrelated or other.

**Conclusions:**

The study provides insights into the changing information-seeking patterns for alpha-gal, indicating growing awareness and interest. Alpha-gal search volume is increasing at a rapid rate. Understanding specific questions and concerns can help health care providers and public health educators to tailor communication strategies. The Google Trends Supercharged-Glimpse tool offers enhanced features for analyzing information-seeking behavior and can be valuable for infodemiology research. Further research is needed to explore the evolving prevalence and impact of alpha-gal syndrome.

## Introduction

Food allergy is a growing public health concern. The global burden of food allergy is increasing, with 220 million individuals worldwide estimated to be affected by at least 1 food allergy [1-3]. In the United States, it is estimated that 26 million, or about 6.2%, of US adults and children have a food allergy [4]. Food allergies can induce a hefty economic burden on individuals and families due to lost labor, productivity, out-of-pocket costs, and opportunity costs [5] and can decrease the quality of life of patients in impacted physical health and mental health [6,7].

One somewhat unconventional food allergy on the rise is the alpha-gal allergy, also known as alpha-gal syndrome, red meat allergy, or tick bite meat allergy [8]. Alpha-gal syndrome is a type of allergy that is characterized by an immune reaction to the carbohydrate molecule galactose-alpha-1,3-galactose (alpha-gal), which is found in most mammalian or “red meat” [9]. This immune reaction can result in a variety of symptoms including hives, swelling of the face or facial features, shortness of breath, abdominal pain, gastrointestinal issues, anaphylaxis, or even fatality [10]. Whereas conventional food allergies usually involve an immediate immune response, alpha-gal allergy is characterized by the delayed onset of symptoms (ie, 3-8 hours following exposure) [8,9,11,12]. This delayed response, compounded with the varying clinical presentation, can make the diagnosis of the allergy challenging.

The alpha-gal allergy stands out not only due to its delayed nature but also because of the unconventional way most individuals develop the allergy. In most cases, it is thought that transmission occurs through the bite of a tick, which injects alpha-gal into the bloodstream. This sensitizes individuals to the molecule and can lead to an immune response when consuming mammalian meat [13]. Alpha-gal cases have been reported worldwide and are associated with various tick species. In the United States, however, growing evidence suggests that alpha-gal allergy is primarily linked to lone star ticks [14]. The connection between alpha-gal allergy and lone star ticks was initially described in 2011 [15] and has since been supported by subsequent studies [12,13,16-18]. Recent evidence from a case-control study further strengthens this association, revealing significantly higher odds of tick exposures among individuals with alpha-gal syndrome compared to controls [16].

Lone star ticks are traditionally found in the Southeastern region of the United States. However, the geographic range of lone star ticks has expanded to the Northeast [19] and Midwest in part due to climate change and sensitivity to microclimate conditions [18]. Indeed, exposure to and cases of alpha-gal allergy are also expanding beyond the Southeastern United States [20]. Recognition and general awareness of alpha-gal allergy are growing [21], but health care providers’ knowledge of the connection between lone star ticks and alpha-gal allergy may be lagging. A recent survey conducted among clinicians in Illinois revealed a lack of awareness regarding the link between lone star tick bites and alpha-gal allergy, as well as limited familiarity with diagnostic testing for the condition [22].

Because there remains considerable variation in health care providers’ knowledge of the condition [23], patients often play a critical role in driving their own diagnoses [24]. As 1 study reported, there is often a lengthy amount of time between symptom onset and diagnosis, with patients visiting multiple health care settings and receiving numerous referrals before obtaining the correct diagnosis [23]. In general, it is likely that many patients rely on finding their own sources of information to learn about the allergy and potentially self-diagnose it before receiving an official diagnosis.

The alpha-gal syndrome is not a reportable or notifiable condition and was only granted a separate International Classification of Diseases *10th Revision* (*ICD-10*) code for diagnostic identification in 2022. Therefore, little data exist on the prevalence of the allergy [8]. In the absence of incidence or prevalence data, infoveillence and infodemiology techniques can be used to help describe the overall population interest in the topic of alpha-gal [25], as well as correlations with potentially related search terms. Many studies over the past decade have used Google Trends as a tool to explore patterns in health information–seeking behavior [26,27]. In fact, 1 prior study using Google Trends found a high correlation between alpha-gal and lone star tick, including a general upward trend in relative search volume (RSV) [21]. Data were examined from 2004 to 2019, but it is likely that both awareness and diagnosis of the allergy have grown in the past 3 years [20].

Further, new tools have emerged that offer enhanced features for analyzing various aspects of information-seeking behavior. One such tool is Glimpse, which is an extension app of Google Trends [28]. Google Trends has been both praised and criticized for reporting all searches as RSVs [29]. RSV is a query share of a search term within a specific geography and time range, which is then normalized to the highest point of popularity within that time period [26]. A notable drawback of this approach is that the absolute volume is not provided; therefore, there is no way to track an estimated number of queries for a particular topic [30]. Because the RSV is indexed to the highest point of popularity for a term within a time period, reproducibility of research results can be challenging [26,29]. To address this issue, the Supercharged-Glimpse extension offers an estimate of the absolute search volume, as well as a dashboard of additional information such as related search terms. The absolute volume numbers are overlayed on the traditional RSV index graph. This tool could be useful for understanding the specific content topics and themes that people search for health information.

Additional quantification of the changing volume of information seeking for alpha-gal can provide valuable evidence regarding shifts in awareness levels and potential changes in alpha-gal prevalence. Moreover, analyzing the evolving information-seeking patterns for both alpha-gal and lone star ticks can offer insights into the changing public interest across different geographic areas. Finally, health care providers and public health officials could benefit from understanding the topics that are most relevant to their patients and the public and adjust their communication strategies for alpha-gal accordingly. Therefore, using the Google Trends Supercharged-Glimpse extension, our objectives were to (1) describe the basic volume and patterns of information seeking related to alpha-gal; (2) explore further correlations between alpha-gal and lone star ticks; and (3) understand and identify specific questions, concerns, and areas of interest that individuals are searching for in relation to alpha-gal.

## Methods

### Google Trends Supercharged-Glimpse

Google Trends Supercharged-Glimpse is a new web-based tool available as an extension of Google Trends [28]. Glimpse functions in parallel with Google Trends and uses a similar random sampling approach to produce reports containing multiple components of trends in web-based information–seeking behavior. Searches are conducted on the Google Trends platform, with options to specify a particular geographic location and time period (spanning from 2004 to the present day). With the Glimpse extension, estimated search trends over time are provided as an absolute volume, rather than RSV. This allows for direct comparisons of volume from separate terms conducted in separate searches. The extension also produces a list of the highest volume of keywords and questions related to the search term, which is akin to a listing of terms that information seekers use in addition to the main keyword. Reports also contain the RSV for geographic areas from Google Trends.

### Search Strategy

To begin our search, we implemented a methodological framework based upon previous strategies aimed to establish a consistent approach to conducting Google Trends research [31,32] and adhered to a suggested checklist for documenting and reporting our search strategy [26]. We report keywords in italics and the classification of the keyword in parentheses.

Google Trends Supercharged-Glimpse was used to determine search trends for 2 alpha-gal keywords: *alpha gal* (search term) and *meat allergy* (search term). Additional keywords related to alpha-gal were explored (*alpha-gal*, *alpha-gal syndrome*, and *alpha gal syndrome*), but these terms were visibly unstable, indicating a low search volume (with most months returning less than 1000 searches) and lower likelihood of using these terms when seeking information. Because these spelling variations produced very low volumes of searches and because the Supercharged tool does not allow modifiers (eg, “+” to add the terms together), we proceeded with the analysis without aggregating these data [31] but recognized these as potential limitations. We selected *lone star tick* (animal) as a keyword to explore objective 2 and *food allergy* (search term) as a broad comparison term. All keyword searches were compared with and without the use of quotations and produced similar results in terms of volume. Data were limited to the United States due to the context of the alpha-gal and lone star tick exploration, and the time frame ranged to include all available data (from January 1, 2004, to March 1, 2023). All keyword searches were conducted separately, without combining keywords. Similar to Google Trends, the Glimpse extension performs a sampling approach to estimate the absolute search volume. As with all sampling approaches, there is a degree of variability each time the data are queried. Further, there is a degree of caching that occurs; therefore, the same search conducted in a short time period (eg, within 10 minutes) may have identical numerical results. To produce a better estimate of the absolute volume of searches for our selected search terms, data were collected at 2 PM daily for a period of 10 days (March 2-12, 2023). Data for each term were compiled and averaged for search volume over time (absolute volume), related search queries (qualitative lists), and geographic interest (RSV). To demonstrate the overlap and correlation between the traditional RSV index values from Google Trends and the absolute volume estimates from the Supercharged-Glimpse extension, the averaged absolute volume and the averaged RSV index values for *alpha gal* (search term) are plotted in Figure S1 in Multimedia Appendix 1. Absolute volume over time for all terms is presented in the *Results* section.

### Statistical Analysis

#### Objective 1: Trends Over Time

Time series analyses were used to assess the trends in all 4 keywords over the study period. A quasi-Poisson regression was fit to account for the overdispersion of the count outcome variables. The regression used a penalized spline on year to account for long-term trends in these various allergies and related searches over time. We opted to use a penalized spline because the data appeared to be nonlinear in the raw data plots. Using penalized splines on the year allowed for the flexibility to account for the complex patterns seen in the raw data plots while not overfitting the model. The seasonality of web-based searches is likely to mirror the seasonality of cases of disease [31]. Therefore, we expected to see seasonal variation in search patterns for all 4 search terms, and indeed, this is what was revealed in graphing the monthly search volumes. There are seasonal variations in tick behaviors and tick-host encounters, which both peak during the warm season [33,34]. Additionally, food-related anaphylaxis has been found to increase during the warm season due to oral allergies (eg, pollen-food allergy syndrome) [35]. Therefore, a binary variable was created to indicate the warm season (May-September), which was then controlled via a linear term to account for seasonal trends. Google trends data for rare diseases can be influenced by the media [29]; therefore, the dates of national media stories on alpha-gal were identified from 2012 through 2023, and this (binary variable for the months that contained national media coverage) was controlled using a linear term. The predicted absolute search volume values are shown in the *Results* section. The average annual percent change (AAPC) in search volume was calculated based on this adjusted model for all 4 terms [36].

#### Objective 2: Alpha-Gal and Lone Star Tick

While our original intent was to describe how information-seeking behavior for both *alpha gal* and *lone star tick* changed geographically and temporally from 2004 to 2022, initial data extraction efforts revealed that the RSV geographic metrics were unstable and unreliable from 2004 to 2013. This is likely due to a lower volume of searches conducted during this time period, which would increase sampling variability. To produce more stable and reproducible results for the geographic analysis, the geographic interest index values were queried and exported in 3-year time period increments (2014-2016, 2017-2019, and 2020-2022) for the *alpha gal* (search term) and *lone star tick* (*animal*) keywords. Choropleth maps were produced. The distribution of the data was found to be nonnormally distributed (Shapiro-Wilk test *P* values <.05 for all variables); therefore, a Spearman correlation matrix was generated to examine geographic and temporal correlations for *alpha gal* and *lone star tick* using the RSV index values for the 3 time periods (2014-2016, 2017-2019, and 2020-2022).

#### Objective 3: Content Analysis

A conceptual content analysis was performed on the related search query data for alpha-gal. Lists from the 10 separate days were examined and found to be nearly identical. Lists were then combined and duplicates were removed, leaving a total of 371 related searches. All 371 related search queries contained the words “alpha gal” either before or after the additional keywords (eg, *alpha gal hives*). For the qualitative content analysis, an inductive coding strategy was first implemented to identify overarching themes based on the content of the data itself. We identified 10 main themes: diet, diagnosis or testing, treatment, medications or contraindications of medications, symptoms, tick related, specific sources of information and locations, general education information, alternative words for alpha-gal, and unrelated or other. Two authors (JR and NJ) independently coded the list of 371 words based on the main concept themes. The results from the coders were compared, and discrepancies were resolved for a final theme designation. This process was repeated within each theme to further identify theme-based subcategories of interest. The total number of subthemes identified was 41. The frequency of keywords in each search theme and theme-based subcategories was compiled and described. All analyses were performed using SAS (version 9.4; SAS Institute) and R Studio (R Foundation for Statistical Computing).

### Ethical Considerations

All data were publicly available and unidentifiable search engine metadata. Data are not used at the individual level and do not involve human subjects; therefore, institutional review board approval was not required for this study.

## Results

### Time Series Analyses

**Figure 1:**
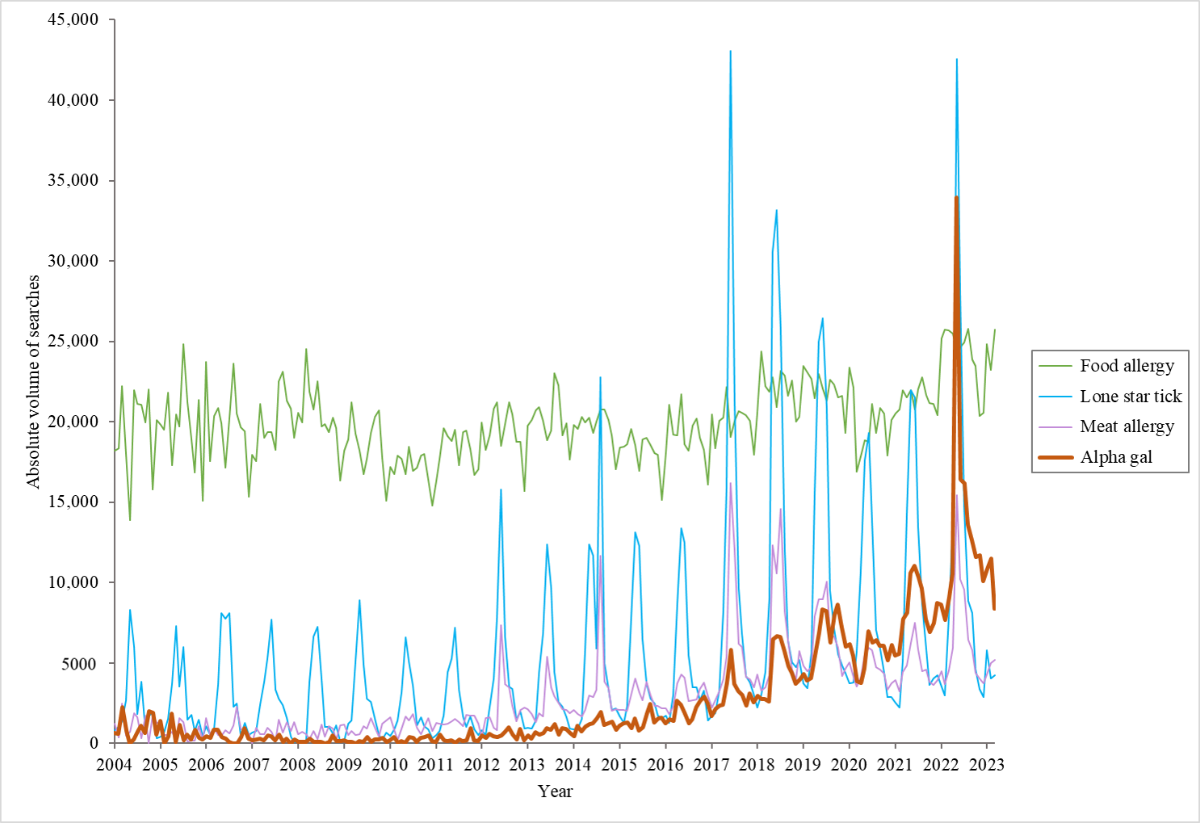
Absolute search volume trends for *alpha gal* (red), *food allergy* (green), *lone star tick* (blue), and *meat allergy* (purple), 2004-2023.

**Figure 2:**
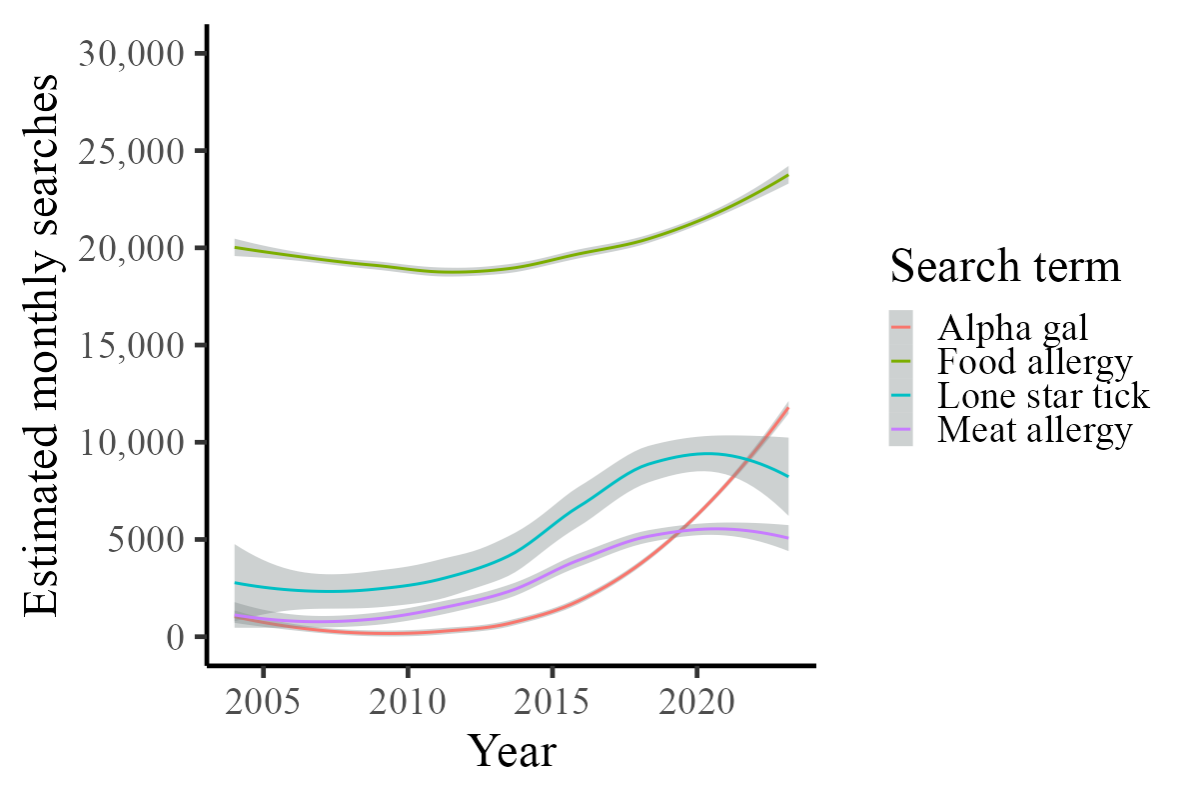
Estimated number of monthly searches for *alpha gal* (red), *food allergy* (green), *lone star tick* (blue), and *meat allergy* (purple) after adjustment for long-term trends, season, and media reports. The mean number of searches for each term is shown by a solid line, and 95% CIs are shaded around each respective line.

The average absolute search volumes from 2004 to March 2023 were plotted for *alpha gal*, *meat allergy*, *lone star tick*, and *food allergy* (Figure 1). The comparison of the traditional RSV index values and the absolute volume estimates demonstrated near-perfect overlap, with a Spearman correlation coefficient of ρ=0.99 (*P*<.001; Figure S1 in Multimedia Appendix 1). In the time series analysis, the estimated absolute search volume values over time were nonlinear for all 4 search terms (Figure 2). The binary indicator for the warm season was a significant predictor for all 4 search terms. While all 4 search terms demonstrated this significant seasonal trend, the magnitude of the warm season term was greatest for *lone star tick* and lowest for *food allergy.* Media coverage was only a significant predictor for *alpha gal*, *lone star tick*, and *meat allergy*. National media coverage of alpha-gal did not significantly impact *food allergy* searches. Based on the expected search volumes after adjusting for seasonality and media events in the time series analysis, the AAPC from 2004 to 2022 was 18.76%, 10.87%, 7.52%, and 1.09% for *alpha gal*, *meat allergy*, *lone star tick*, and *food allergy*, respectively. Interestingly, search trend volume began to noticeably increase starting around 2015 for *alpha gal.* From January 2015 to January 2022, the AAPC was 33.78%, 9.23%, 7.34%, and 2.45% for *alpha gal*, *meat allergy*, *lone star tick*, and *food allergy*, respectively. The estimated total increase during this time period was 627%, 81%, 61%, and 25% for *alpha gal*, *meat allergy*, *lone star tick*, and *food allergy* searches.

### Alpha-Gal and Lone Star Tick

**Figure 3:**
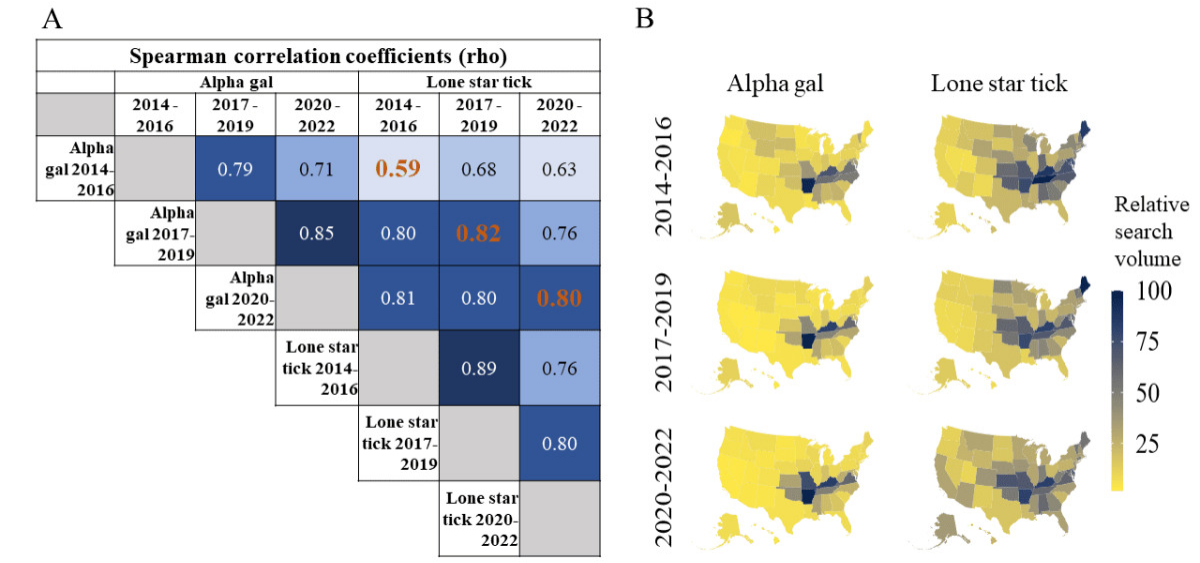
Spatial and temporal correlations between alpha gal and lone star tick information seeking over time. A. All Spearman ρ correlations for state-based RSV values were significant at the *P*<.001 level. Orange values indicate the 3 primary correlations of interest. B. Correlations can be further visualized in the choropleth maps. Dark blue states on the map indicate high RSV values. RSV: relative search volume.

Choropleth maps of the RSV indices for *alpha gal* and *lone star tick*, as well as the Spearman correlation coefficient for each time period, are presented in Figure 3. All correlations were significant at *P*<.001. State RSV indices for *alpha gal* and *lone star tick* were moderately correlated in 2014-2016 (ρ=0.59) but became more strongly correlated in the latter 2 time periods (ρ=0.82; ρ=0.80). These correlations can be visualized by the noticeable geographic overlap observed over time between states with a high information-seeking interest in *alpha gal* and *lone star ticks*.

### Content Analysis

#### Overview

**Figure 4:**
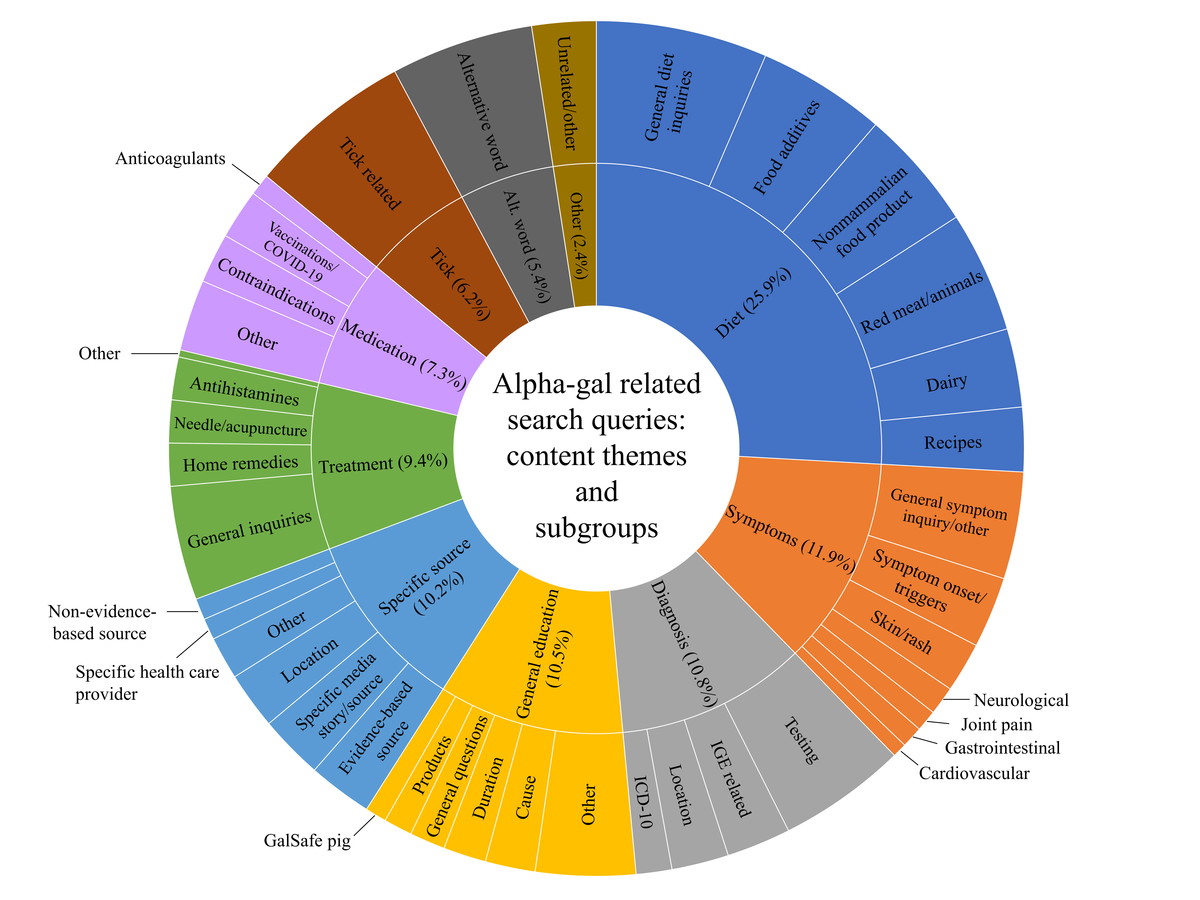
Content analysis of related search queries. Ten primary themes were identified, and multiple subcategories within the themes revealed diverse information-seeking behavior. ICD-10: International Classification of Diseases 10th Revision.

The content analysis revealed 10 overarching themes (Figure 4). Of the 10 overarching themes, 7 were further divided into subcategories.

#### Diet

The most diverse search theme was diet (96/371, 25.9%), which could be further divided into 6 subthemes. The most popular subtheme was composed of general inquiries (eg, *what foods to avoid with alpha gal?* and *what foods are safe with alpha gal?*), followed by food additives (eg, *gelatin*, *glycerin*, and *magnesium stearate*), red meat (ie, *specific mammal inquiries*), nonmammalian food options (eg, *ostrich*, *eggs*, and *turkey*), dairy products (eg, *butter*, *milk*, and *cheese*), and searches for recipes.

#### Symptoms

Symptom-related searchers accounted for 11.9% (44/371) of the total related search queries. General symptom–related questions or inquiries (eg, *what are the symptoms?*) were most prominent, followed by symptom onset or triggers (eg, *reaction time*, *onset*, and *exercise*). The other 5 specific subtheme symptoms that emerged were skin or rash (7/44, 15.9%), neurological (4/44, 9.1%), joint pain (3/44, 6.8%), gastrointestinal (3/44, 6.8%), and cardiovascular (2/44, 4.5%) symptoms.

#### Diagnosis

Diagnosis-related searchers accounted for 10.8% (40/371) of the total related searches, including 4 subthemes of general searches for testing (eg, *blood test* and *test results*), specific searches for IgE (eg, *IgE numbers* and *levels*), specific laboratories that perform testing, and searches for *ICD-10* codes.

#### General Education

General education and information seeking accounted for 10.5% (39/371) of the related searches. This theme was further broken down into causes (eg, *what is the cause of alpha gal?*, *is alpha gal genetic?*, *is alpha gal contagious?*), duration (eg, *is alpha gal permanent?*, *does alpha gal go away?*), general questions (eg, *what is alpha gal?*), products, information seeking on the knockout pig, and other (eg, *alpha gal and COVID-19*, *alpha-gal while pregnant*, and *prevalence*).

#### Specific Sources

Specific sources of information or location accounted for 10.2% (38/371) of searches. Around 24% (9/38) of these were media-specific searches (eg, *alpha-gal radiolab* and *nytimes*), 24% (9/38) were evidence-based sources (eg, *NIH*, *UpToDate*, *CDC*, and *Mayo Clinic*), and 21% (8/38) were location or place specific (eg, *Kentucky*, *Lynchburg* *VA*, and *UVA*). Other subgroups included nonevidence-based sources, specific clinician or health care provider searches, and others.

#### Treatment

Treatment-related searches accounted for 9.4% (35/371). Most searches were general (eg. *how to treat alpha gal* and *is it curable?*), while others were specifically related to acupuncture, antihistamines, and home remedies (eg, *natural remedies* and *essential oils*).

#### Medication

Medication or contraindication-related searches accounted for 7.8% (27/371) of related searches. Roughly 25% (7/27) of these searches were vaccine related (eg, *flu shot* and *COVID-19 vaccine*). About 25% (7/27) were inquiries about medications known to contain alpha-gal (eg, heparin), while the majority (37%, 16/27) were general inquiries on what medications were necessary to avoid (eg, *alpha gal in medications*, *drugs to avoid*, *alpha gal medication list*, and *alpha gal* and *anesthesia*).

## Discussion

### Principal Findings

To our knowledge, we believe this is one of the first studies to investigate absolute search volume trends over time using the Google Supercharged-Glimpse extension app. Our analysis revealed several important trends and patterns regarding public interest and information seeking for alpha-gal allergy.

First, over the past several years, the volume of searches for the alpha-gal allergy is significantly increasing at a rapid rate. After adjusting for seasonality and national media stories, the overall increase for *alpha gal* searches over time was at least 6 times that of the other search terms. Interestingly, *alpha gal* search term volume surpassed that of *meat allergy* in 2019, which likely indicates an increase in the awareness of the correct terminology for the allergy. Further, while *lone star tick* and *meat allergy* search term volumes seemed to level off from 2020 to 2022, *alpha gal* search term interest continued to grow. National media stories were found to have a significant impact on the search volume for *alpha gal*, *meat allergy*, and *lone star tick* terms, highlighting the role that media can play in engaging public interest and awareness of these topics.

Geographic popularity information-seeking for the term *alpha gal* did not vary greatly over time and remained concentrated in the southeast regions of the United States. While this finding was similar to a study using 2019 data [21], it was somewhat unexpected. Diagnoses of alpha-gal syndrome have occurred in other geographic regions including the Northeast and Midwest [20], and cases are increasing in those areas [8]. It is possible that this finding might be reflective of how the RSV index is generated. States with larger populations could have a greater absolute volume of interest, but this might not be reflected in the RSV index. There were no states throughout time with an RSV index of 0, which indicates that people are seeking information for the alpha-gal allergy in all 50 states. This finding is similar to laboratory-based studies that show documented cases in most states [20].

Geographic searches for the terms *alpha gal* and *lone star tick* strongly overlap. States have similarly ranked RSV indices for both *alpha gal* and *lone star tick* terms, and these have strengthened over time. Of note, geographic search interest in lone star ticks appears to be expanding. Search interest maps from 2014 to 2016 and 2017 to 2019 strongly overlap with a 2016 study that documented expansions of the lone star tick range [19]. Furthermore, search interest maps from 2020 to 2022 show a similar overlap with predicted lone star tick habitat expansion models developed in 2021 [37]. It is possible, therefore, that future interest and prevalence maps for alpha-gal may expand in similar ways. This serves as a forewarning, highlighting the urgent need to expand education efforts for both the general public and health care providers.

Finally, people are seeking a broad array of topics related to alpha-gal. The largest subgroup of content searched for in conjunction with alpha-gal was tick related. Encouragingly, national media stories and evidence-based sources comprised almost 50% of the specific sources that people were searching for. Unsurprisingly, diet was the largest theme, and interest in concealed sources of exposure and food additives remains a large concern [11]. The ubiquitous presence of animal products in food, medications, and other products can make it extremely difficult for individuals to know if what they ingest is safe [8]. Given the challenges of identifying safe foods and products, there is a clear need for improved food and product labeling. Additionally, information seeking for specific symptoms highlighted the broad array of ways that alpha-gal can manifest within an individual. Symptom variety remains one of the major reasons alpha-gal remains underdiagnosed. Health care professionals should not only be familiar with the most commonly identifiable symptoms of alpha-gal such as urticaria or anaphylaxis [18] but also be knowledgeable about symptom manifestations like joint pain [8], gastrointestinal symptoms [12,18,38], dizziness, or heart palpitations [10].

### Limitations

There are several limitations to this study. First, we were only able to capture information seeking on 1 search engine platform. Second, absolute search volume estimates were not available geographically, and the RSV geographic data were unstable when examined yearly. We chose to sacrifice the granularity of a year-by-year analysis in favor of more reliable results. Third, the absolute volume was also not provided for each of the related searches. Therefore, the proportional composition of the content analysis themes represents the diversity within the theme, not necessarily the popularity of the theme. It is possible that tick-related searches encompassed the majority of the volume of the related alpha-gal searches, but we cannot identify this based on the data. Finally, at the time of the initial data analysis and submission of this study, alpha-gal syndrome was only available as a search term and not a disease. Since that time, the term has become searchable on the Google Trends platform as a designated syndrome. Absolute search volume trends over time for both *alpha gal* (search term) and *alpha-gal syndrome* (syndrome) are remarkably similar, indicating there is likely direct overlap between these 2 searches. It is unknown to what degree the addition of this condition as a designated syndrome may affect the volume reported for the *alpha gal* (search term) in the future.

### Conclusions

Information seeking for alpha-gal syndrome is rapidly increasing. Geographic overlap with lone star tick searches might suggest future expansions in alpha-gal interest and prevalence, thereby emphasizing the urgency for increased education efforts. The diverse range of topics and symptoms searched for highlights the ongoing challenges faced by individuals affected by alpha-gal. However, the content and subthemes identified can serve as a valuable guide to facilitate public health outreach and effective patient-physician communication.

## Appendix

Multimedia Appendix 1 Comparison of absolute search volume and relative search volume (RSV) index over time for *alpha gal* (search term).

## Abbreviations

AAPC: average annual percent change
ICD-10: International Classification of Diseases 10th Revision
RSV: relative search volume

## Data Availability

The data sets generated and analyzed during this study are available from the corresponding author on reasonable request.
