# Supplementary figures and images for: Emerging Trends in Information-Seeking Behavior for Alpha-Gal Syndrome: Infodemiology Study Using Time Series and Content Analysis
Source: J Med Internet Res. 2024 May 8;26:e49928. doi: 10.2196/49928 (PMC11112475; doi:10.2196/49928)

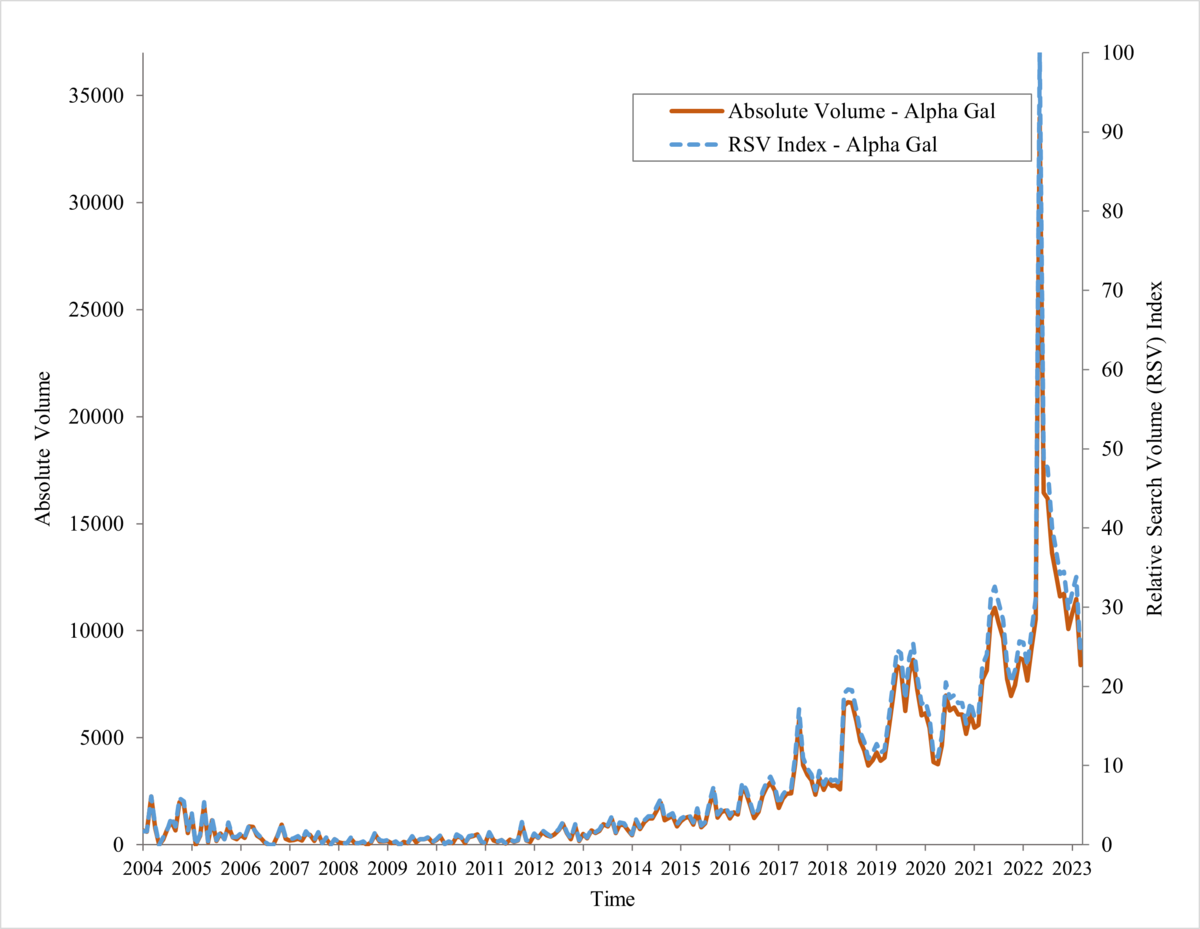

Supplement: Multimedia Appendix 1 [file jmir_v26i1e49928_app1.png]
